# Taking a mixed-methods approach to collision investigation: AcciMap, STAMP-CAST and PCM

**DOI:** 10.1016/j.apergo.2021.103650

**Published:** 2022-04

**Authors:** Omar Faruqe Hamim, Shahnewaz Hasanat-E-Rabbi, Mithun Debnath, Md Shamsul Hoque, Rich C. McIlroy, Katherine L. Plant, Neville A. Stanton

**Affiliations:** a Department of Civil Engineering, Bangladesh University of Engineering and Technology, Dhaka, 1000, Bangladesh; b Accident Research Institute, Bangladesh University of Engineering and Technology, Dhaka, 1000, Bangladesh; c Department of Civil Engineering, Ahsanullah University of Science & Technology, 141 & 142, Love Road, Dhaka, 1208, Bangladesh; d Human Factors Engineering, Transportation Research Group, University of Southampton, Southampton, UK

**Keywords:** Accimap, STAMP-CAST, PCM

## Abstract

Recently, ergonomics and safety researchers have turned their attention towards applying combinations of sociotechnical methods rather than using single methods in isolation. In the current research, a mixed-method approach combining two systems-based methods, Accimaps and the Systems Theoretic Accident Model and Process - Causal Analysis using Systems Theory (STAMP-CAST), and one cognitive approach, the Perceptual Cycle Model (PCM), were employed in analysing a rail-level crossing incident in Bangladesh. Each method was applied individually to investigate the collision, and interventions were proposed corresponding to incident events at different risk management framework levels. The three methods provided different perspectives of the whole picture, together identifying an array of contributory factors. The complementary nature of these methods aided in proposing a comprehensive set of safety recommendations, thereby demonstrating the benefit of a mixed-method approach for collision investigation in low-income settings.

## Highlights

• Combining systems-based and cognitive methods for road-rail collision investigation.
• Interventions are proposed corresponding to events at different system levels.
• Complementary nature of methods used brings benefits in collision investigation.

## 1 Introduction

Unintentional non-compliance with rail-level crossings by road users causes significant safety issues around the world. This is due, at least in part, to an incomplete understanding of the many complex underlying sociotechnical systemic and cognitive factors, and their interactions, that lead to such incidents (Salmon et al., 2017; Stanton et al., 2017). The International Union of Railways (UIC) reported that in 2018, of all recorded, significant railway incidents in Europe, 14.7% occurred at rail-level crossings, and involved 18% of all victims (UIC Safety Report, 2019). In Bangladesh, 235 rail-level crossing incidents resulted in 244 fatalities and 228 injuries in 2018 (The Daily Star, 2019). According to Bangladesh Railway, fatalities at rail-level crossings across the country are still rising, and over eighty percent of rail-level crossings in the country, both authorized and unauthorized, remain unprotected (The Daily Star, 2019).

Many road crash investigation systems have been criticized by researchers due to a lack of alignment with contemporary models of collision causation, and an excessive focus on driver behaviours (Salmon et al., 2016). It has been argued that the interactions among the many different actors of a system can be better understood using sociotechnical systems thinking approaches (Hulme et al., 2021; McLean et al., 2021). Following calls for the application of systems thinking approaches to the road safety domain, a growing body of research can be found that tackles the evolving nature and increasing complexity of road transport (e.g., Salmon, et al., 2016; Parnell et al., 2017; Li et al., 2019). This even extends to the road-rail interface domain, where research has demonstrated that a systems approach can enable the identification of effective design improvements to rail level crossings (Read et al., 2013). The very large majority of this research has been undertaken in high-income settings; however, a number of applications of such methods in low- and middle-income countries (LMICs) have recently been seen (e.g., McIlroy et al., 2019a, 2020a; Hamim, 2020; Hamim et al., 2020a, 2020b).

Alongside advances in systems-thinking and road safety, psychology and the cognitive ergonomics field continues to be useful in helping us understand why collisions occur. It has been argued that the Perceptual Cycle Model (PCM: Neisser (1976), described in more detail below), with its underpinning schema theory, is useful in explaining the mechanisms underlying failures in human performance (Norman, 1981; Plant and Stanton, 2012; Debnath et al., 2021). Whereas systems methods, with their view of factors hierarchically abstracted from the immediate collision environment, represent a macro approach to collision analysis, cognitive ergonomics can be considered as a micro approach in its focus on the end user cognition and behaviour. It has been argued that a combined application of these macro and micro models can work holistically in collision investigation (Salmon and Read, 2019), with the integration of methods bringing new dimensions to road safety research. This is the focus of the current research. Specifically, we assess the suitability of a combined application of two systems methods, namely Accimaps (Svedung and Rasmussen, 2002) and Causal Analysis based on the Systems Theoretic Accident Model and Process (STAMP-CAST; Leveson, 2004), with the cognitive PCM approach for the investigation of a road-rail collision, with a particular focus on traffic safety in low-income countries, where the road trauma burden is most felt but the safety research community least active.

The three methods differ in some important ways. The Accimap method is based on systems theory (Rasmussen, 1997) and maps out an event's contributory factors on a hierarchical diagram. The STAMP-CAST approach is also based on a hierarchical description of a system but focusses on failures in control and feedback; hence, a control structure model is produced. As described above, the PCM is an approach based on the schema theory of human cognition and action and is represented using cyclical psychological models. Ours is not the first to assess the combination of methods. For example, multiple ergonomics methods have been assessed in terms of the differences in their outputs (e.g., in terms of results, reliability and validity; Salmon et al., 2012; Stanton et al., 2009). Stanton et al. (2005) introduced the concept of using integrated methods to analyse performance in complex sociotechnical systems from multiple perspectives, and Salmon and Read (2019) demonstrated the utility of applying multiple systems-based methods in combination for road safety studies; however, the utility of a combination of systems approaches (macro-level) and psychological approaches (micro-level) to analyse the same problem space has not yet been investigated.

In Bangladesh, rail-level crossing incidents are not officially documented by the concerned authorities (e.g., Bangladesh Railway or Police), hence there is a lack of quality collision data. There is also a dearth of academic literature on road-rail safety in Bangladesh, and in LMICs more widely. Motivated by these needs, this research analyses a fatal collision between a van and a train occurring at a rail-level crossing. This particular incident was chosen for analysis as it drew significant national level attention from the news media, government officials, and the general public, and is generally representative of the types of road-rail collision events that have been reported in the media in Bangladesh.

The aim is, therefore, to demonstrate the utility of a mixed-method approach through the application of Accimaps, STAMP-CAST, and the PCM to the analysis of a rail-road collision, and their support for the identification of suitable safety interventions in Bangladesh. Although our aim is to provide evidence for the benefit of the approach in all settings, particular attention is paid to low-income settings, where such collision investigation techniques are very rarely seen, and where end-user blame dominates discussions of responsibility for road trauma even more so than it does in high-income settings.

## 2 The incident

In early 2015, a road-rail collision incident was investigated by a team at the Accident Research Institute (ARI) of Bangladesh under the guidance of the then Director of that institute (one of the authors of the current article). Witness interviews were performed by two of the current authors (as members of the investigation team) immediately after the incident occurred, and a series of photographs were taken at the incident spot. The incident occurred in a restricted warehouse area with other road users being absent, resulting in a lack of witnesses; only the guard stationed at the rail-level crossing could provide information regarding the incident in addition to the van driver and locomotive master involved in the collision. Based on the information retrieved from the interviews and an examination of the photographs of the incident site, the following reconstruction of events was developed.

At about 1:15 p.m. on the 29th of December 2014, six passengers died and more than 20 were injured in a collision between a passenger train and a van at a rail-level crossing near the Kamalapur Inland Container Depot (ICD) run by Chittagong Port Authority (CPA). Controlled by the Ministry of Shipping, the CPA transfers imported products to the Kamalapur ICD from Chittagong port via the Bangladesh Railways. Kamalapur ICD has two parts divided by rail tracks. A wide road, solely used by the vehicles transporting goods to and from the depot, crosses the railway tracks via a rail-level crossing within the ICD area. The incident occurred when the train was passing through the ICD area with compartments full of passengers. In addition to travelling inside the passenger carriages, people were sitting or standing on the roofs and railings of the engine and other compartments.

Although there were no traffic sign-markings, a flashing light traffic signal was present along the rail-lines to warn road vehicles of oncoming trains; this was not working. There were no lights at the roadside for crossing road vehicles, and no physical boom barriers were present; instead, two bamboo barriers were placed to block the tracks. L-shaped solid boundary walls erected along the road edge as well as along the railway tracks obstructed the vision of both the van driver and the train master. The train master did not blow the train's whistle before passing the level crossing. Upon seeing the train approaching, it is assumed that the van driver thought that he would be able to cross before the train arrived, hence did not stop, rather sped up, even though an Ansar guard (law enforcing agencies vested with public security duties) stationed for guarding the rail-level crossing hand signalled him to stop. Due to the absence of a road divider for channelizing traffic flow, the van driver was able to manoeuvre to the right, occupying the wrong lane, so that he could cross the tracks prior to the train's arrival; however, the van had only partially crossed the level crossing upon collision with the train.

**Fig. 1:**
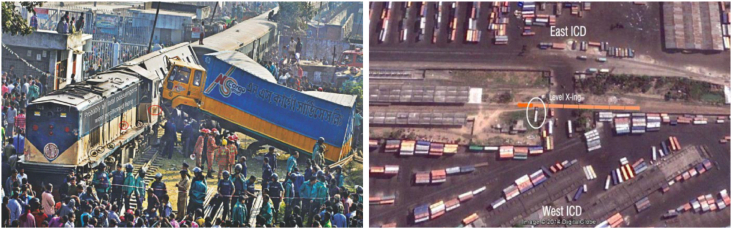
Incident location (taken from the investigation report of ARI, 2015).

The train engine hit the rear side of the covered van, rotated it by 180°, and dragged it about 50 yards away from the incident spot after smashing it against a boundary wall pillar. While the van was being dragged by the train, the train's engine and first compartment were severely damaged by the van's bumper; this caused derailment of the train. The van became stuck in between the train and an outer railway track, and as a result, the van became anchored against the outer railway track and started to slide along the train body, colliding with those passengers who were standing on the engine railing. Two of the standing passengers died at the scene and significant damage was incurred to both the train and the van. Photographs of the incident locations are presented in Fig. 1.

## 3 Methods and analyses

### 3.1 Accimap

Accimaps (Svedung and Rasmussen, 2002) graphically represent the systemic causal factors involved in collisions and typically use the following six Risk Management Framework (Rasmussen, 1997) levels: government policy and budgeting; regulatory bodies and associations; local area government planning and budgeting; company management, technical and operational management; physical processes and actor activities; and equipment and surroundings. In this research, we use an extended version that includes two additional levels at the top of the hierarchy, namely international committees, and national committees (Parnell et al., 2017).

**Fig. 2:**
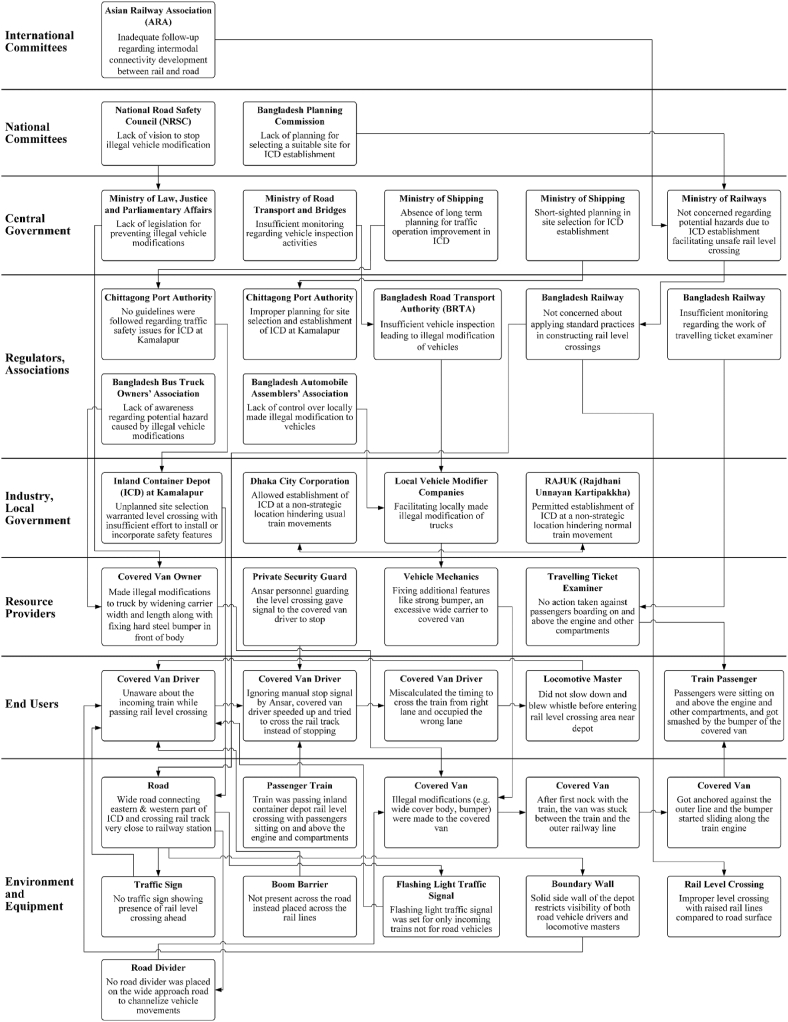
Combined Actor Map and AcciMap of the Kamalapur rail-level crossing incident.

In preparing the Accimap, the ARI investigation report was used along with news reports from digital and printed media, photographs of the incident site, and at-scene photo coverage archived at the ARI. The Bangladesh Actor Map developed by McIlroy et al. (2019a) aided in identifying the actors relevant to specific system levels, acting as a starting point for model development. After collecting all the available data, the authors convened to discuss the incident and the web of contributing factors. One of the authors first developed a draft combined Actor Map and Accimap; this was then reviewed by the other authors and disagreements resolved through further discussion. The final combined Actor Map and Accimap was validated by the Principal Investigator of the investigation committee; this is presented in Fig. 2.

The combined Actor Map and Accimap presented in Fig. 2 identifies the key parties that are potentially involved in influencing the collision. Many related actors and their interconnections have been uncovered spanning across eight levels of the system, starting from ‘environment and equipment’ at the lowest level up to ‘international committees’, the highest level which identifies the contribution (or lack thereof) of each relevant actor. The events, failures, decisions, and actions are shown in the boxes with relationships between them indicated by the arrows.

### 3.2 STAMP-CAST: Systems Theoretic Accident Model and Process – Causal Analysis using systems theory

STAMP-CAST (Leveson, 2004) is an incident-analysis technique based on the premise that system failures (in our case, a collision) result from insufficient control or enforcement of safety constraints, and from inadequate feedback mechanisms. STAMP-CAST attempts to identify dysfunctional interactions between each level of a system (Leveson, 2004). The first stage of constructing a STAMP-CAST model involves identifying the actors and organizations responsible for controlling the system. Control and feedback mechanisms between different system levels are then included in order to show what controls are enacted down the hierarchy and what information about the status of the system is sent back up the hierarchy.

**Fig. 3:**
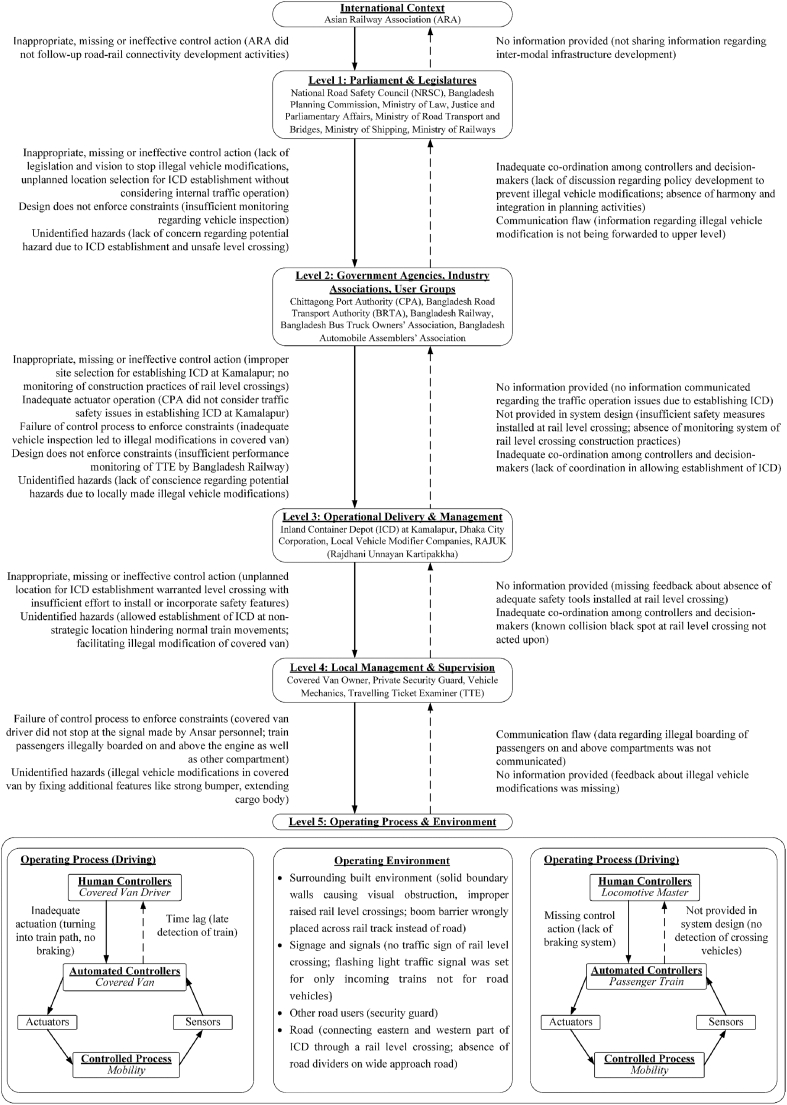
Kamalapur rail-level crossing incident STAMP-CAST model.

The STAMP-CAST model for the incident under analysis was constructed by identifying the key actors involved across different levels of the system, again drawing on the Bangladesh Actor Map (McIlroy et al., 2019a). After graphically plotting the responsible organizations at various levels of the system, the relevant control and feedback constraints were annotated on the control structure, with solid downward arrows representing control mechanisms and dashed upward arrows representing feedback mechanisms. Each of the systemic elements identified within the control structure were then classified according to the classification of flawed control (Leveson, 2004). This taxonomy functions by evaluating the potential contribution of each control and feedback loop, listing the potential failures; these are presented in Table 1. These are general factors applicable at each level of the control structure, but the interpretations of the factor will differ based on its application at different levels. The initial draft STAMP-CAST model was reviewed by the other authors and any discrepancy in the draft model was discussed and refined accordingly. Again, the final STAMP-CAST model was validated by the Principal Investigator of the original investigation committee; this is presented in Fig. 3.

**Table 1:** Taxonomy of control flaws resulting in possible hazards.

Control Flaws	Possible Hazards
1. Inadequate or inappropriate control actions issued by the controller	i) Failure to identify possible hazards
	ii) Constraints not enforced by the design of control process
	ii) Inadequate co-ordination among controllers and decision-makers
2. Inadequately executed control actions	i) Communication flaws
	ii) Inadequate actuator operations
	iii) Time lags
3. Missing or inadequate feedback	i) Non-existent feedback processes in the system's design
	ii) Communication flaws
	iii) Time lags
	iv) Incorrect information provided in the system
	v) Lack of co-ordination among controllers and decision-makers

### 3.3 The perceptual cycle model (PCM)

**Fig. 4:**
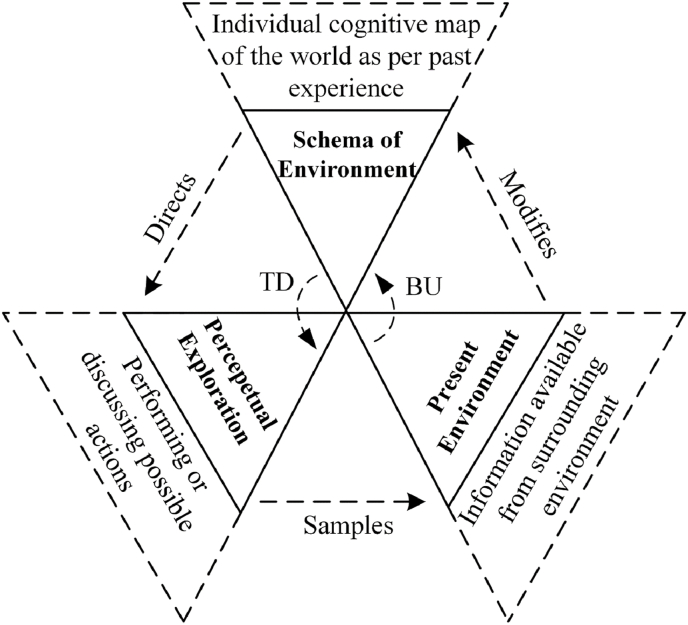
Perceptual cycle model (Neisser 1976). TD: Top down, BU: Bottom up.

Neisser (1976) perceptual cycle model (Fig. 4) represents the view that human cognition is reciprocally related to a person's interaction with the world. Internally held mental templates (i.e., schema) help a person to understand situations and anticipate certain types of information; these schemas direct a person's behaviour for seeking relevant information about the world in an interpretative manner, and experience ultimately modifies and updates schema while influencing further interaction with the environment (Plant and Stanton, 2012). The flow of information occurs in both top down (TD) and bottom up (BU) directions (Plant and Stanton, 2016).

Three perceptual cycle models for the incident were developed using descriptions in police reports, information from print media and online portals, and witness interviews. These corresponded to different involved individuals’ experiences: the van driver, the locomotive master, and the security guard. The analysis included the events and activities from moments before the arrival of the van at the rail-level crossing up to the occurrence of the collision. The likely thought processes of the individuals involved in the occurrence of the crash were constructed based on the interviews of the security guard, the van driver, the locomotive master, and the train passengers involved in this incident. The interviewees were individually asked questions about the factors and events that influenced their decisions. Responses were considered in terms of how they corresponded to other interviewee comments and to findings from the investigation of the incident location.

The constructed thought processes were thematically coded based on the categories of perceptual cycle model, i.e., schema, action, and world. Norman (1981) taxonomy of schema-related errors were then applied to the resulting PCM diagrams. This taxonomy proposes that a schema controls a sequence of actions and is associated with three possible types of error, as presented in Table 2.

**Table 2:** Classification of schema errors.

Taxonomy	Type of Errors
1. Failure in formation of intention	i) Mode errors: failure in properly classifying a situation resulting from an inappropriate formation of intention
	ii) Description errors: failure in correctly specifying acts in the absence of required situational information
2. Faulty activation of schema	i) Capture errors: stronger sequence taking control under similar action sequences
	ii) Data-driven activation errors: external events activate schemas
	iii) Loss of activation errors: schemas losing activation after being activated
	iv) Forgetting an intention: continuing with action sequence even after forgetting the intention
	v) Errors in task sequencing: jumbling orders, skipping, or repeating steps
3. Failure to trigger appropriate schema	i) Spoonerisms: event components are reversed
	ii) Blend errors: combination of components from competing schemas causing faults

**Fig. 5:**
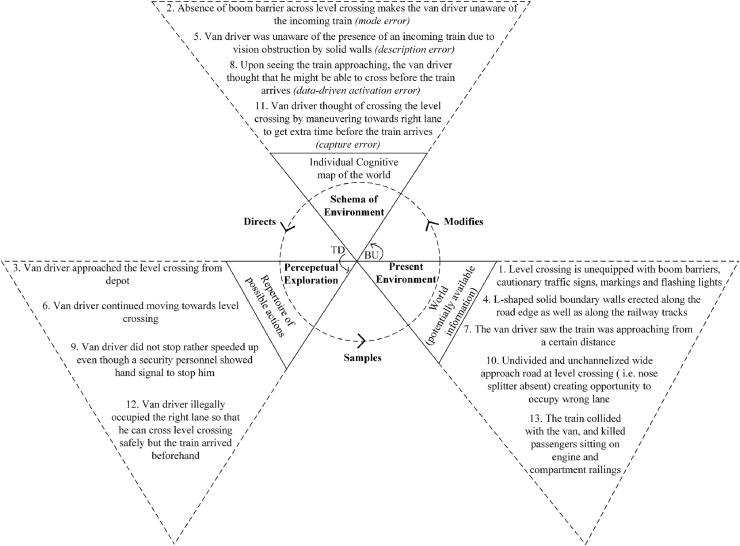
Van driver PCM diagram of the Kamalapur rail-level crossing incident.

**Fig. 6:**
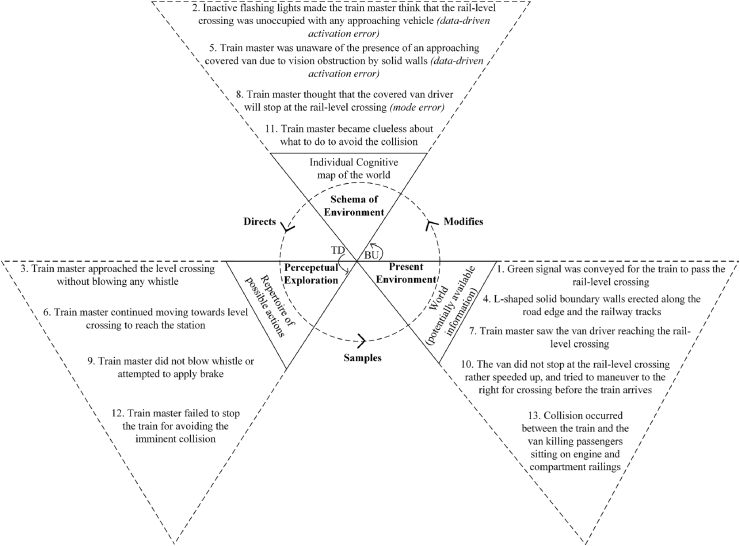
Train master PCM diagram of the Kamalapur rail-level crossing incident.

**Fig. 7:**
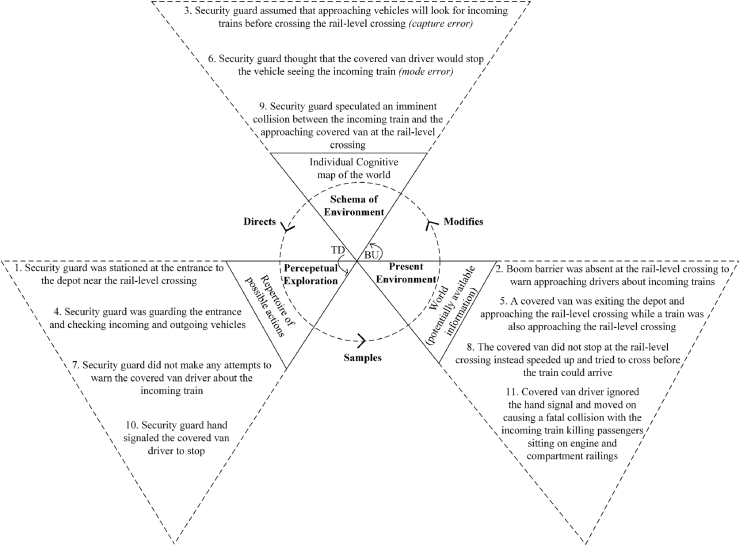
Security guard PCM diagram of the Kamalapur rail-level crossing incident.

Initially, draft PCM diagrams were created by one of the current authors; these were then discussed with the other authors. Disagreements were resolved collaboratively, following which PCM elements were classified according to the error taxonomy and depicted in the diagrams using italic fonts. The final PCM models are presented in Fig. 5, Fig. 6, Fig. 7. Fig. 5 is discussed in detail, Fig. 6, Fig. 7 are summarised.

The PCM diagram in Fig. 5 is based on the cognitive processes of the van driver. It can be observed from the ‘world’ section that the rail-level crossing was equipped with neither boom barriers, cautionary traffic signs, markings, nor flashing lights (point number 1 in Fig. 5). This led to mode error in formation of a schema; thus, the van driver unaware of the incoming train (point 2). As the van driver approached the rail-level crossing (3), the L-shaped boundary walls erected along the road edge and railway tracks (4) obstructed his view of the train; this lead to a description error in formation of the schema, again resulting in the driver being unaware of the train's presence (5). As the van and the train continued moving towards the rail-level crossing (6), the van driver saw the train approaching from a distance (7). He judged that distance to be sufficient for him to be able to cross before the train's arrival (8); this represents a data-driven activation error (i.e., the faulty analysis of the train's distance and speed led to the action). The van driver therefore sped up, ignoring the security guard's hand signal to stop (9). The undivided and unchannelized approach road (10) contributed to his confidence thinking that he might get extra time to cross (11) by manoeuvring towards the right lane (12); this led to a capture error. In combination, these errors in formation and activation led to the van driver's illegal occupation of the right lane with the goal of passing the level-crossing before the arrival of the train. Together, all of these events created the necessary and sufficient conditions for the collision to occur.

The PCM diagram presented in Fig. 6 represents the train master's processing cycle. The green signal and the L-shaped boundary walls (obstructing his vision) could have made him suppose that no vehicle was approaching, representing a data-driven activation error. He therefore neither attempted to brake nor blew the whistle. Upon seeing a van approaching, the train master made a mode error in anticipating that the van would stop.

Fig. 7 presents the PCM diagram detailing the cognitive processes of the security guard stationed close to the rail-level crossing. Due to the absence of a boom barrier, the security guard presumed that any approaching vehicle would look for incoming trains before passing (a common practice among drivers); this represents a capture error in schema activation. The simultaneous approach of the train and van made the security guard think that the van driver would stop; this represents a mode error in schema formation. The security guard's hand signal was too late and was not heeded.

## 4 Proposing recommendations

To compare each method's contribution to developing a safer overall system, road safety intervention recommendations were developed based on the findings of each of the individual analyses presented above. These were then combined and are presented in Table 3, with the right-most column indicating from which method the safety intervention arose (note that many recommendations arose from more than one analysis). The recommendations developed were then classified in terms of the Risk Management Framework level (RMF, i.e., the Accimap levels) to which they applied.

**Table 3:** Recommendations corresponding to events identified in the Accimap, STAMP-CAST and PCM analyses, mapped according to the six RMF levels.

Level	Incident Event	Recommendation	Method Source
International Committees	Asian Railway Association (ARA) did not follow-up road-rail connectivity development activities	Develop a monitoring unit to follow-up road-rail connectivity development activities of partner countries	Accimap, STAMP-CAST (control)
	Information regarding inter-modal infrastructure development was not shared	Information regarding inter-modal infrastructure development must be communicated with ARA	STAMP-CAST (feedback)
National Committees	Lack of legislation and vision to stop illegal vehicle modification from National Road Safety Council (NRSC)	NRSC needs to develop a monitoring cell and arrange safety campaigns to demotivate road users from using illegal vehicle modifications	Accimap, STAMP-CAST (control)
	Unplanned location selection for ICD establishment without considering internal traffic operation by Bangladesh Planning Commission	At planning stage, all relevant issues must be considered before selecting site location of major structural establishment	Accimap, STAMP-CAST (control)
Central Government	Lack of legislation for preventing illegal vehicle modifications	Ministry of Law, Justice and Parliamentary Affairs should formulate law and order to stop illegal vehicle modifications	Accimap
	Insufficient monitoring regarding vehicle inspection by Ministry of Law, Justice and Parliamentary Affairs, and Ministry of Road, Transport and Bridges	Monitoring cells need to be developed within responsible ministries to oversee vehicle inspection activities	Accimap, STAMP-CAST (control)
	Short-sighted planning by Ministry of Shipping while selecting site for ICD establishment	Ministry of Shipping should focus on long-term planning for new site for ICD shifting	Accimap
	Lack of concern regarding potential hazard due to establishment of ICD and unsafe level crossing from Ministry of Railways, and Ministry of Shipping	Potential hazards need to be addressed before establishing major ventures	Accimap, STAMP-CAST (control)
	Lack of discussion regarding policy development to prevent illegal vehicle modifications	Policy developments need to be emphasized to prevent illegal vehicle modifications	STAMP-CAST (feedback)
	Absence of harmony and integration in planning activities	Coordination must be ensured through establishing integration cells among organizations bestowed with planning responsibilities	STAMP-CAST (feedback)
	Lack of information sharing regarding illegal vehicle modifications	Information regarding illegal vehicle modifications must be shared with planning level organizations	STAMP-CAST (feedback)
Regulators and Associations	Improper site selection for establishing ICD at Kamalapur without considering traffic safety issues by Chittagong Port Authority (CPA)	Traffic safety issues must be considered before establishing commercial activity centers by conducting traffic impact assessment (TIA)	Accimap, STAMP-CAST (control)
	Inadequate vehicle inspection by Bangladesh Road Transport Authority (BRTA)	Adequate number of vehicle inspectors need to be appointed by BRTA for properly inspect vehicles	Accimap, STAMP-CAST (control)
	Lack of monitoring regarding rail-level crossing construction practices, and insufficient performance monitoring of travelling ticket examiner by Bangladesh Railway	Construction practices at rail-level crossing and performance of travelling ticket examiner must be strictly monitored by Bangladesh Railway	Accimap, STAMP-CAST (control)
	Lack of conscience from Bangladesh Bus Truck Owner's Association and Bangladesh Automobile Assembler's Association regarding potential hazards due to locally made illegal vehicle modification	Mass consciences need to be raised regarding potential hazards due to locally made illegal vehicle modification	STAMP-CAST (control)
	Information regarding the traffic operation issues due to establishment of ICD was not communicated	Traffic operation issues raised due to establishment of ICD must be communicated with upper level authorities	STAMP-CAST (feedback)
	Insufficient safety measures were installed at rail-level crossing	Adequate safety measures (e.g., traffic signs and signals, boom barrier, road divider etc.) must be installed at rail-level crossings to keep road users informed about potential hazards	STAMP-CAST (feedback)
	Absence of monitoring system of rail-level crossing construction practices	Monitoring system of rail-level crossing construction practices must be developed	STAMP-CAST (feedback)
	Lack of coordination in allowing establishment of ICD	Inter-department coordination need to be ensured before allowing major establishments	STAMP-CAST (feedback)
Industry and Local Government	Dhaka City Corporation and RAJUK (Capital Development Authority) allowed establishment of ICD at a non-strategic location hindering usual train movements	Before allowing any establishment to be constructed, research needs to be conducted to identify potential safety risks/threats by conducting traffic impact assessments (TIA)	Accimap, STAMP-CAST (control)
	Facilitating illegal modifications of covered van by local vehicle modifier companies	Illegal vehicle modifications by local vehicle modifier companies must be punished with high amount of penalties	Accimap, STAMP-CAST (control)
	Unplanned location for ICD establishment warranting level crossing with insufficient effort to install or incorporate safety measures by Inland Container Depot authority at Kamalapur	Well-planned construction at strategic locations with adequate safety features must be ensured by concerned authorities	STAMP-CAST (control)
	Missing feedback about absence of adequate safety tools installed at rail-level crossing	Feedback about absence of adequate safety tools installed at rail-level crossing must be passed on to upper level authorities	STAMP-CAST (feedback)
	Not acting upon known collision blackspot	Known collision blackspots must be improved according to expert advice	STAMP-CAST (feedback)
Resource Providers	Illegal vehicle modifications were made in covered van by fixing additional features like strong bumper, extended cargo body by vehicle mechanics	Strict punishment must be ensured against illegal vehicle modifications	Accimap, STAMP-CAST (control)
	No action taken against passengers boarding on and above the engine and other compartments	Passengers boarding on and above the engine and other compartments should be charged with high fines	Accimap, STAMP-CAST (control)
	Data regarding illegal boarding of passengers on and above compartments was not communicated	Information related to illegal boarding of passengers on and above compartments must be communicated with higher authorities for taking appropriate actions	STAMP-CAST (feedback)
	Feedback about illegal vehicle modifications was not communicated	Feedback about illegal vehicle modifications need to be passed on to higher authorities to take strict actions	STAMP-CAST (feedback)
End users	Covered van driver did not stop rather speeded up even though a security personnel showed hand signal to stop him	Awareness among drivers need to be raised regarding adherence to traffic rules and adequate security forces need to be employed for proper enforcement of traffic laws	Accimap, STAMP-CAST (control), PCM (action)
	Covered van driver miscalculated the timing to cross the train from right lane and occupied the wrong lane	Drivers need to be trained on safe use of rail crossings	Accimap, STAMP-CAST (control), PCM (action)
	Locomotive master could not brake due to lack of adequate braking system	Upgraded trains having sustainable brake systems and equipment need to be used for ensuring enhanced safety	Accimap, STAMP-CAST (control)
	The train collided with covered van and killed passengers sitting on engine and compartment railings	Passengers must not be allowed to sit on engine and compartment railings and must be made cautious about the safety issue	Accimap, PCM (world)
	Covered van driver was late in detecting the incoming train	Cautiousness while encountering rail-level crossing need to be created among drivers through defensive driving trainings	STAMP-CAST (feedback)
	Locomotive master did not get information regarding the vehicle crossing	A sensor-based system for detecting the presence of road vehicles must be placed at rail-level crossings for informing locomotive master about crossing vehicles	STAMP-CAST (feedback)
	Covered van driver and train locomotive master were unaware of the presence of each-other due to vision obstruction by solid walls	Instead of allowing the construction of vision obstructing solid walls, see-through fence type walls should be constructed at rail-level crossings	PCM (schema)
	Upon seeing the train approaching, the covered van driver thought that he might be able to cross before the train arrives	Drivers need to be provided with defensive driving training for evaluating crossing scenarios	PCM (schema)
	Covered van driver thought of crossing the level crossing by manoeuvring towards right lane to get extra time before the train arrives	Strict punishment with high penalties need to be enforced for occupying wrong lane	PCM (schema)
Equipment and Environment	Wide road connecting eastern & western part of ICD and crossing rail track very close to railway station	ICD should be shifted at a distant place from the station so that no rail level crossings are warranted	Accimap
	Road connecting eastern and western part of ICD through a rail-level crossing was not facilitated with road dividers	Wide approach roads at rail-level crossings must be provided with road dividers	Accimap, STAMP-CAST (control), PCM (world)
	Illegal modifications (e.g., wide cover body, bumper) were made to the covered van	Prohibiting illegal body modification should be strictly enforced	Accimap
	Traffic sign of rail-level crossing, and flashing light traffic signal were absent	Traffic sign and signals need to be adequately facilitated at rail-level crossings	Accimap, STAMP-CAST (control), PCM (world)
	Boom barriers were wrongly placed across railway tracks instead of road	Boom barriers need to be properly placed across road	Accimap, STAMP-CAST (control), PCM (schema)
	Rail-level crossing was improperly raised	Rail-level crossing must be constructed at-grade with connecting roadway	STAMP-CAST (control)
	Solid side wall of the depot restricts visibility of both road vehicle drivers and locomotive masters	For a clear line of sight, solid side wall needs to be replaced with grill-type wall	Accimap, STAMP-CAST (control), PCM (world)

**Fig. 8:**
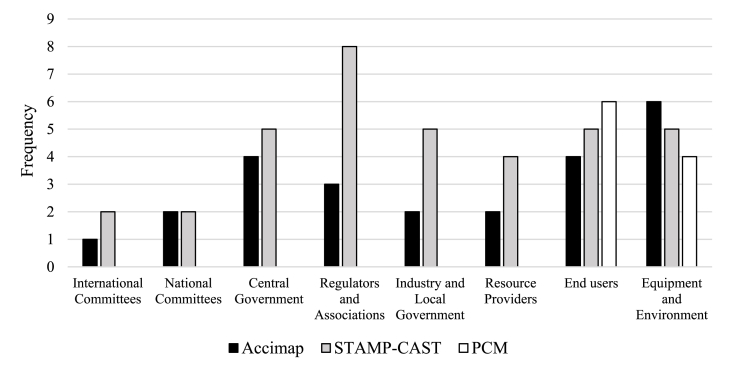
Summary of the recommendations mapped against system levels.

In total, STAMP-CAST gave rise to 36 recommendations, Accimap to 24 recommendations, and the PCM analysis to 10 recommendations. The frequency of recommendations proposed at different levels of the system, separated by analysis method, is shown in Fig. 8. The PCM analysis supported recommendations for interventions at only the bottom two levels, as would be expected given its focus on human performance. Also as expected, given their higher-level focus, both Accimap and STAMP-CAST analyses supported the proposal of recommendations at the upper levels as well as at the lower levels of the system.

## 5 Discussion

It has previously been argued that complex multi-faceted systems are better understood and improved through the application of multiple methods (Stanton et al., 2005; Page, 2016; Salmon and Read, 2019). As such, this paper's aim was to provide evidence for the benefit of using ergonomics methods at both the systems (macro) and cognitive (micro) levels through the use of Accimaps, STAMP-CAST, and the PCM approach to the analysis of an incident in a complex road-rail transport system in Bangladesh.

Comparing the methods, Accimap is taxonomy-free, hence gave flexibility in the identification of actors and their interconnections across system levels. With its associated taxonomy, STAMP-CAST aided in identifying the failures in feedback and control mechanisms. PCM, on the other hand, provided a comprehensive explanation of schema-driven human behaviour, something lacking in both STAMP-CAST and Accimap. Many of the recommendations presented in Table 3 stemmed from all three methods; however, each method also provided unique contributions. It is therefore clear that the combined use of systems-based approaches and cognitive-based approaches increases the level of comprehensiveness of event description and recommendation generation.

Because of the existence of a failure taxonomy in STAMP-CAST, it was the only model that supported identification of interventions related to feedback mechanisms between system levels. Even though the Accimap can include both control and feedback mechanisms, its lack of taxonomy makes this method more dependent on the judgement of the analyst (Stanton et al., 2019). This can result in the non-identification of feedback failures, as evident from the analysis presented above. We therefore echo Salmon and Read (2019) recommendation that both are used together.

Further to the STAMP-CAST and Accimap analyses, the PCM analyses added details to incident event descriptions, thus expanding upon the countermeasures proposed by the other two methods. Indeed, the micro-level of analysis provided benefit in a different but complimentary manner, as it helped shed light on the impact of (for example) the obstruction of the train and van drivers' line of sight, and the van driver's misjudgement of the train's speed and his decision to drive in the opposing lane to get across the crossing. The combination of the different models, therefore, yielded additive benefits to the analysis and to the proposal of countermeasures. This overcomes some of the drawbacks of traditional approaches to road traffic collision investigation. Typically, these do not consider the higher system factors that shape outcomes. The use of systems methods, with their focus on broader contributory factors, direct the conversation away from the end user and their physical environment. Moreover, despite the end-user focus characteristic of traditional investigation methods, the inclusion of a cognitive ergonomics model also adds benefit. Traditional methods rarely consider the psychological aspects that may have contributed to a collision, at least not explicitly. The use of the PCM brings out these factors. Therefore, the combination of micro and macro ergonomics methods can bring significant benefit to collision investigation and the subsequent proposal of road safety intervention recommendations.

The analysis above focussed on the events leading up to the collision, rather than on the aftermath. Regarding the PCM, our use of the model focussed on the decision-making processes of actors immediately involved in the incident at that moment, and in the moments leading up to the collision. It would be possible to perform PCM analyses on the decision-making activities of emergency responders, but this was not the focus of the current work. The Accimap does lend itself to post-event analysis, with some advocating its use for the analysis of emergency responding (Salmon et al., 2014; Hamim et al., 2020a, 2020b). As a developing country, the need of improved rapid response recovery system cannot be denied (Hamim et al., 2020b), hence, use of a mixed-method approach for post-collision analysis represents a valuable avenue for further study.

The non-compliance of drivers with rail-level crossing controls has been identified as a notable component in road-rail collisions, especially in high income countries (Lenné et al., 2011; Salmon et al., 2013; Larue et al., 2020); however, the scenario differs in low- and middle-income countries. In many cases, adequate safety measures are found to be non-existent. This was certainly the case with the incident analysed above. For example, Saccomanno et al. (2007) found that boom barriers increase the safety performance of rail-level crossings. Each of the methods applied above highlighted the absence of boom barriers, as well as the lack of flashing traffic signal lights and the train whistle, as influential factors. Each method also drew attention to the presence of the L-shaped solid walls along the road edge and railway tracks. Such construction characteristics are uncommon in settings with well-established infrastructure standards and procedures. The presence of passengers hanging onto the outside of a train carriage body also highlights a difference between high- and low-income settings. This practice is largely unthinkable in high income countries, yet is very common in densely populated, low-income countries. This contributed to the large number of fatalities and injuries in the collision analysed above. Further, illegal vehicle modifications, an improperly raised rail-level crossing, use of old (now obsolete) locomotives, and unplanned constructions, were all highlighted as contributory factors in this incident, factors that one would generally not expect to find in high-income settings.

The combined application of Accimap, STAMP-CAST, and PCM helped generate a variety of safety recommendations across the system levels. For reasons of brevity, we cannot discuss every recommendation presented in Table 3 in detail; however, discussion of some examples is merited. One such example is the recommendation that upgraded trains be equipped with sustainable (i.e., long-lasting, and fixable) brake systems. In Bangladesh, locomotives more than 50 years old (with braking systems of the same age) are still being used (Digital Logistics Capacity Estimations, 2018). Although the train brakes were not reported to have malfunctioned during the incident, it is to be emphasized that using rolling stock of such an age would not be recommended from a safety perspective.

The installation of a sensor-based system for detecting the presence of road vehicles at all rail-level crossings was also recommended. This would be used for informing the locomotive master about crossing vehicles so that they can whistle to warn the incoming vehicles and to apply brakes in advance, something that could reduce the consequences of collision should one occur. Further, in order to support informative and unimpeded visual scanning of the environment, barriers through which one can see (e.g., chain-link fencing) should be installed in place of solid walls. This would aid in avoiding a collision in the event of a malfunctioned warning light. Given that intersections are more hazardous than any other type of road segment (in terms of collision statistics; Briz-Redón et al., 2019), from a cognitive perspective, drivers could be provided with driving training that supports the safe evaluation of typical rail-crossing scenarios (and other intersections). From a systems perspective, all existing rail-level crossings should be treated to meet international level crossing standards. Such practice is absent in Bangladesh, and in many other low-income settings.

Any multi-method approach requires a relatively high level of resource input in terms of training, quality and quantity of data, and analyst time, yet there is evidence for their benefit. Read et al. (2017) combined Accimap analyses, components of the Event Analysis of Systemic Teamwork (EAST), Cognitive Work Analysis (CWA), Hierarchical Task Analysis (HTA), and driving simulation studies in the development of various rail-level crossing interventions in Australia. Their work highlighted the benefits of the many-model approach, building on work reported in Salmon and Read (2019). The analysis presented in this paper shows that the combined application of Accimap, STAMP-CAST, and PCM covers both the sociotechnical systems and cognitive branches of ergonomics science. Notwithstanding the additional analyst time, some of the resources required (e.g., data collection) are common to all methods, and all analysis procedures require methodological efforts at the desktop level. We argue that the combination used above therefore strikes a balance between resource usage and analysis coverage, and can be considered as the “Lite” approach mentioned by Salmon and Read (2019). An initial Accimap analysis provides a comprehensive map of contributory factors and the relationships between them. Following this, STAMP-CAST can aid in describing the control and feedback mechanisms present in (or absent from) the system. Finally, the PCM analysis then gives more detail on the cognitive activities of the end-users involved.

Although the lack of an associated taxonomy makes the Accimap flexible, it also makes it more difficult for the analyst to identify failures. To address this shortcoming, Salmon et al. (2020) analysed 23 Accimaps to produce a set of 79 generic factors contributing to system failure across domains. This taxonomy can be used to support analysts to structure Accimaps in future investigations; however, it is still in its infancy, and requires additional attention before it can be fully accepted. Hence, combining Accimaps with STAMP-CAST is justified as the taxonomy of STAMP-CAST is well established. Nevertheless, where both Accimap and STAMP-CAST fall short is in the detailed treatment of end-user cognition. With its relatively simple schema-action-world taxonomy, and its cyclical model of processing, the PCM readily supports the analysis of such behavioural and cognitive factors. A more extensive PCM taxonomy has been developed by Plant and Stanton (2017) for investigating failure in the aviation domain. This could be applied to the road and rail domains with very little adaption, though work would be required to confirm this. In short, the three methods complement each other.

Currently, the overwhelming majority of research on the application of integrated suites of ergonomics methods has been performed in high-income settings (e.g., Thoroman and Salmon, 2020; Salmon and Read, 2019).. To our knowledge, the current study represents the first to test the applicability of a multi-method approach in a low-income setting, where the road trauma burden, and therefore the requirements for such research, are considerably higher (WHO, 2018). Although many low-income countries share some road transport characteristics (e.g., relatively low motorisation rates, highly congested cities, aging vehicle fleets, etc.), there is significant variety in traffic culture (e.g., Nordfjærn et al., 2014), system design (McIlroy et al., 2019a), and end-user behaviour (e.g., McIlroy et al., 2019b; 2020a; 2020b) across countries. As such, repetition and expansion of this work in different low- and middle-income settings would be a highly valuable addition to the traffic safety literature.

## 6 Limitations of the study

In developing the Accimap, STAMP, and PCM models, the main source of information acquisition and validation lies in police investigation reports, news media reports, and witness interviews. Due to the lack of comprehensive police collision reports, a post-collision investigation exercise was carried out by a group of collision researchers that included two of the current authors, and a series of interviews were conducted with the victims, witnesses, the train driver, and the truck driver. As with all activities of these kinds, it is possible that different analysts or investigators may have elicited additional details from the individuals interviewed. This is accepted as a limitation of the current study and is one that is common to all collision investigation and analysis exercises.

Analysts with varying experience and skill in applying sociotechnical methods can lead to different results. Similar to the point made about data collection, therefore, it is possible that different analysts would produce slightly different Accimap, STAMP, and PCM models. Again, this is a potential limitation of all such analysis methods; however, this is somewhat mitigated by expert validation. This also applies to the recommendations made; the subjective nature of the recommendations proposed necessitates validation by subject matter experts. Finally, collision data acquisition is a challenge in low-income settings; this was a major reason in why this particular incident was chosen for analysis, as the initial investigation was carried out by two of the current authors and it was extensively reported on in the popular media. We must accept that analysing only one incident, in one setting, will give a limited view of the applicability of multiple methods to road traffic collision investigation in LMICs. We therefore consider the research presented here as a first step, one that should be followed up by extensive application of the methods across a variety of settings.

## 7 Conclusions

This paper presented a multi-method approach to collision investigation with the aim of providing a more comprehensive explanation of an incident and offering greater insights in order to generate more comprehensive countermeasure recommendations. A rail-level crossing incident was investigated with the combined application of the Accimap, STAMP-CAST and PCM methodologies, thereby offering both a cognitive ergonomics, micro level perspective, and a sociotechnical system, macro level analysis. Initially, the insights extracted from the Accimap analysis provided the basis for a STAMP-CAST model; this detailed the control and feedback mechanisms present in the system, and the failures therein. Then followed an end-user level analysis using the PCM model, offering insight into the decision-making activities of the main actors involved. Although mixed-method endeavours can be resource intensive, it has been argued that adopting multiple perspectives produces benefits that outweigh the associated costs.

## Declaration of competing interest

The authors declare that they have no known competing financial interests or personal relationships that could have appeared to influence the work reported in this paper.
